# Using remote sensing to map larval and adult populations of *Anopheles hyrcanus *(Diptera: Culicidae) a potential malaria vector in Southern France

**DOI:** 10.1186/1476-072X-7-9

**Published:** 2008-02-26

**Authors:** Annelise Tran, Nicolas Ponçon, Céline Toty, Catherine Linard, Hélène Guis, Jean-Baptiste Ferré, Danny Lo Seen, François Roger, Stéphane de la Rocque, Didier Fontenille, Thierry Baldet

**Affiliations:** 1 Territories, Environment, Remote Sensing and Spatial Information Joint Research Unit (UMR TETIS), Maison de la Télédétection, 500 rue J.-F. Breton, 34093 Montpellier Cedex 5, France; 2 French Agricultural Research Center for International Development (CIRAD), Epidemiology and Ecology of Animal Diseases Unit, Baillarguet Campus, 34398 Montpellier Cedex 5, France; 3 Institut de recherche pour le développement (IRD), UR016, Caractérisation et contrôle des populations de vecteurs, 911 avenue Agropolis, BP 64501, 34394 Montpellier cedex 5, France; 4 Department of Geography, Université Catholique de Louvain, 3 place Pasteur, 1348 Louvain-la-Neuve, Belgium; 5 Entente interdépartementale pour la démoustication (EID) Méditerranée, 165 avenue Paul Rimbaud, 34184 Montpellier cedex 4, France

## Abstract

**Background:**

Although malaria disappeared from southern France more than 60 years ago, suspicions of recent autochthonous transmission in the French Mediterranean coast support the idea that the area could still be subject to malaria transmission. The main potential vector of malaria in the Camargue area, the largest river delta in southern France, is the mosquito *Anopheles hyrcanus *(Diptera: Culicidae). In the context of recent climatic and landscape changes, the evaluation of the risk of emergence or re-emergence of such a major disease is of great importance in Europe. When assessing the risk of emergence of vector-borne diseases, it is crucial to be able to characterize the arthropod vector's spatial distribution. Given that remote sensing techniques can describe some of the environmental parameters which drive this distribution, satellite imagery or aerial photographs could be used for vector mapping.

**Results:**

In this study, we propose a method to map larval and adult populations of *An. hyrcanus *based on environmental indices derived from high spatial resolution imagery. The analysis of the link between entomological field data on *An. hyrcanus *larvae and environmental indices (biotopes, distance to the nearest main productive breeding sites of this species *i.e*., rice fields) led to the definition of a larval index, defined as the probability of observing *An. hyrcanus *larvae in a given site at least once over a year. Independent accuracy assessments showed a good agreement between observed and predicted values (sensitivity and specificity of the logistic regression model being 0.76 and 0.78, respectively). An adult index was derived from the larval index by averaging the larval index within a buffer around the trap location. This index was highly correlated with observed adult abundance values (Pearson r = 0.97, p < 0.05). This allowed us to generate predictive maps of *An. hyrcanus *larval and adult populations from the landscape indices.

**Conclusion:**

This work shows that it is possible to use high resolution satellite imagery to map malaria vector spatial distribution. It also confirms the potential of remote sensing to help target risk areas, and constitutes a first essential step in assessing the risk of re-emergence of malaria in southern France.

## Background

Vector-borne diseases are particularly influenced by environmental conditions and environmental changes [1,2]. Indeed, arthropod vectors in general, and insect vectors in particular are very sensitive to their environments, which determine their presence, development and behaviour. As a consequence, climatic as well as landscape and land cover factors greatly influence the spatial distribution of vectors and the diseases they transmit [3].

As remote sensing techniques provide valuable information on such environmental conditions [4,5], several studies have used remote sensing imagery to map the distribution of vector species at different spatial scales [6-10]. The objectives of such studies may differ, according to the epidemiologic context. In endemic areas, mainly in tropical and subtropical regions, these vector maps are designed to improve vector control, which is currently one essential method in limiting the burden of important vector-borne diseases such as malaria or dengue fever. For example, studies mapping suitable areas for malaria vectors or disease have been made at the continental scale for Africa [11-13] and at the regional or local scale in regions of Africa [14-18], Central America [19-21] and Asia [22,23]. In disease-free areas, analyzing the link between the environment and potential vector distribution may help evaluate the risk of emergence of the disease [24], and lead to better understanding of the ecology of the invasive vector species [25].

In the recent years, several human and zoonotic diseases have emerged (or re-emerged) and spread in European countries formerly free of these diseases [26,27]. Thus, in a context of climatic and landscape changes, the question of the possible re-emergence of malaria in Europe has to be re-examined [28]. Although several models have predicted a potential increase of malaria in Europe, there is a general agreement that the risk is very low under current socio-economic conditions. However, occasional autochthonous cases recently reported in Italy [29], Spain [30], Germany [31] and Greece [32] highlight the importance of updating the current distribution of the potential European malaria vector as a preliminary "mapping risk" step toward predicting future scenarios.

In metropolitan France, malaria was endemic until the beginning of the 20^th ^century in marshy areas such as the Rhone delta [33]. Malaria disappeared from this area after World War II due to the drying of marshes, growth of livestock, improvement of housing and life conditions, and the use of quinine; the last *Plasmodium vivax *malaria epidemic occurred in 1943 [34]. Nevertheless, autochthonous transmission was suspected in the French Mediterranean coast in 2006 [35], supporting the idea that southern France remains suitable for malaria transmission. Among eight anopheline species described in the Camargue area (Rhone delta), recent entomological surveys identified *Anopheles (Anopheles) hyrcanus *(Pallas) as the main potential malaria vector because of its abundance and anthropophily [36]. Moreover, this species is involved in malaria transmission in Afghanistan [37]. The presence of this species is usually associated with irrigated rice growing areas [38,39], but *An. hyrcanus *larvae were also reported in other biotopes such as reed beds and marshes with *Scirpus *[40]. Recently, a study highlighted the potential of low spatial resolution imagery in providing pan-European maps of the distribution of five *Anopheles *species potentially capable of serving as malaria vectors at a continental scale [41]. As far as we know, however, no study has yet mapped *An. hyrcanus' *presence in Europe.

In this context, the main objective of the study was to determine and quantify the spatial distribution of *An. hyrcanus *larval and adult populations in the Camargue area of the delta of the Rhone River in southern France. A secondary objective was to determine if rice paddies singly explained the distribution of *An. hyrcanus *populations, or if other biotopes may also be favorable to the development of this species. We used high spatial resolution imagery to characterize the land cover and analyzed the link between land cover variables and field data on the presence/absence of *An. hyrcanus *larvae, leading to the definition of a larval index. An adult *An. hyrcanus *index was then derived from the larval index. Field survey data on the abundance of adult *An. hyrcanus *populations were used to validate this index. The method is simple and is designed to be equally applicable for the mapping other mosquito species distributions.

## Methods

### Study area

**Figure 1:**
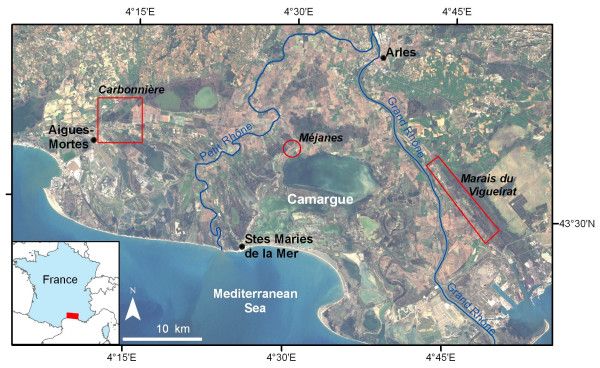
**Study area and location of the field study sites for entomological sampling**. Background: Landsat ETM+ image, 25 October 2001, Eurimage^©^.

The study area is located in the south of France, between 43.33° and 43.73° north and 4.05° and 4.93° east (Figure 1). It encompasses the Rhone river delta and its surroundings. This area has a Mediterranean climate with hot, dry summers and mild, wet winters. Landscape is composed of wet areas (coastal lagoons, marshes, rice fields) and dry areas (agricultural zones, Mediterranean scrubland, forests).

Within this area, two main study sites were chosen to carry out entomological surveys on *Anopheles *species (larvae and adult mosquitoes) [36]. The first one, named "Marais du Vigueirat" is a natural reserve minimally impacted by human activity, and is located in the wet area. In the second one, called "Carbonnière", located between wet and dry areas, human activities are more prevalent and mosquito control is performed against *Ochlerotatus caspius*, a pest mosquito. These control measures have been conducted for 40 years and may impact other mosquito species populations, depending on the period and the places where they are conducted. Both areas present the same variety of biotopes (rice fields, marshes with *Scirpus*, reeds, rush wetlands and meadows). Additional data on larvae were collected in a third site between "Marais du Vigueirat" and "Carbonnière", in a rice paddy area named "Méjanes" (Figure 1).

### Entomological data

**Figure 2:**
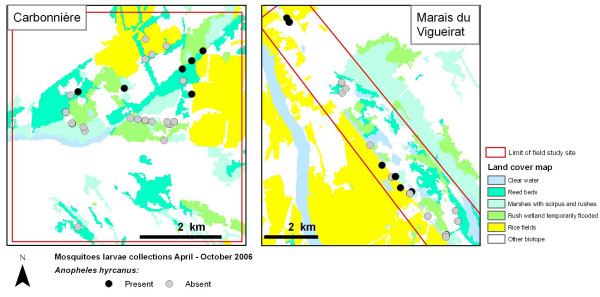
Land cover map of the study sites (Carbonnière and Marais du Vigueirat, Camargue, France) and results of *Anopheles hyrcanus *larvae collection, April – October 2006.

80 potential breeding sites (37 in "Marais du Vigueirat", 41 in "Carbonnière" and two in "Méjanes"), situated in the main biotopes potentially suitable to *An. hyrcanus *larvae, were visited every month from April to October, 2006, except in April and May when only 23 and 52 sites were prospected (due to water management and dryness in summer, some potential breeding sites were dry when visited). This period corresponds to the mosquito season' duration. Mosquito larvae were collected using standard dipping techniques; larvae were stored in absolute alcohol and identified using morphological keys [42]. For each place visited, the presence or absence of water was reported, as well as other characteristics: temperature, salinity and pH, depth, turbidity, presence or absence of organic matter, exposure to the sun, vegetation and biotope. Finally, the presence (of one or more individuals) or absence of *An. hyrcanus *larvae in each site during the whole period (April-October) was derived from all these observations (Figure 2). Spatial autocorrelations of the mosquito larvae samples were analyzed by calculating Moran's I index (ArcGIS Spatial Statistics Tools).

**Figure 3:**
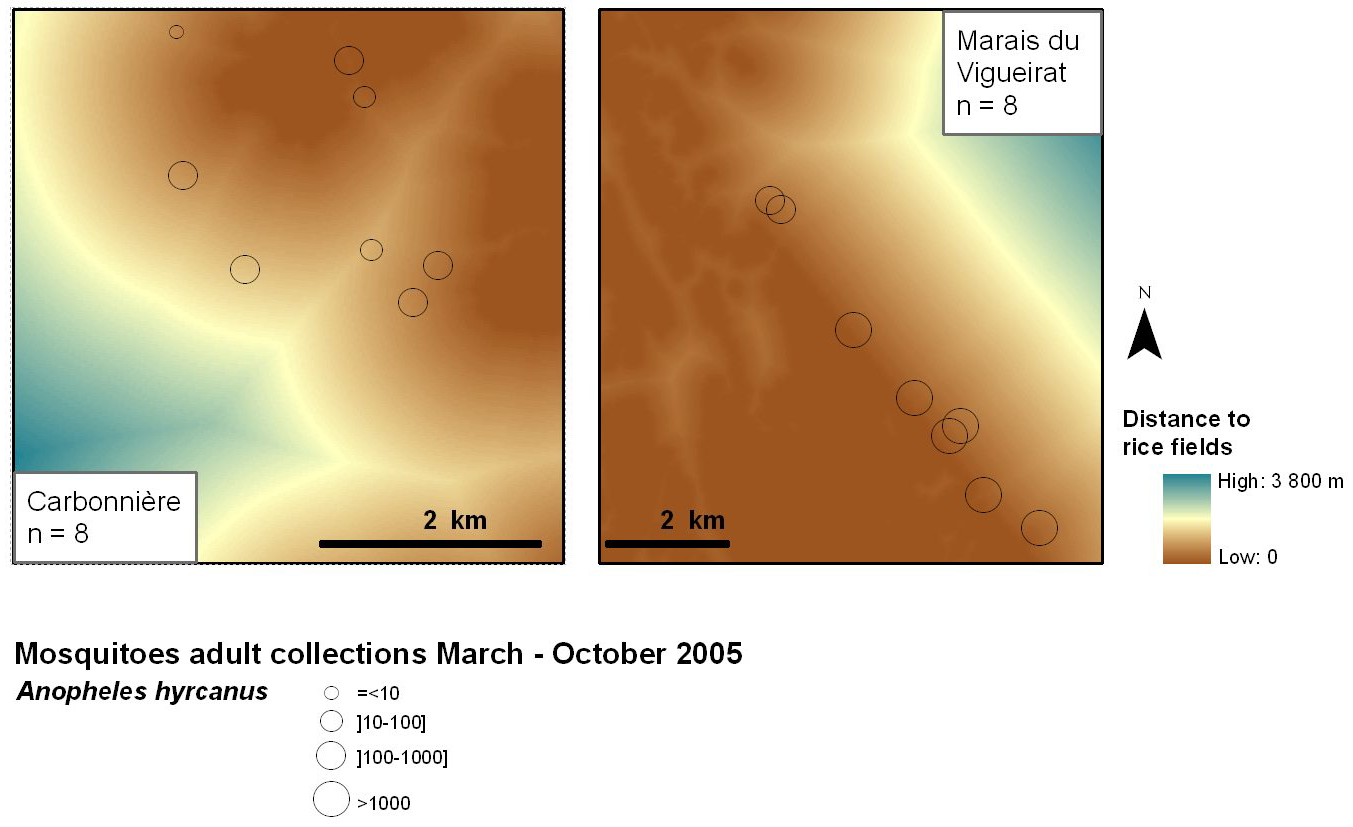
**Results of *Anopheles hyrcanus *adult populations trapping in Carbonnière and Marais du Vigueirat study sites, Camargue area, France, March – October 2005, and distance to the rice fields**. The circles show the maximum number of *Anopheles hyrcanus *adult mosquitoes trapped between March and October. The colour of the background indicates the distance to the nearest rice field (points near to rice fields are red-coloured while distant points are blue-coloured).

Adult mosquitoes were captured from March to October, 2005. CDC-light traps associated with carbon dioxide dry ice were hung in 8 locations in each study site ("Marais du Vigueirat" and "Carbonnière") from 19:00 to 10:00 hours, for two consecutive nights, once every two weeks (512 collections). Mosquitoes were identified using morphological keys [36,42]. The mean number of mosquitoes per trap was calculated using the results of the two consecutive nights. Then the maximum mean number of mosquitoes in the year was computed (Figure 3) to obtain the most abundant capture of the year.

All collections of adult and larval mosquitoes were localized using Global Positioning System (GPS) receiver.

### Environmental data

To describe the environmental characteristics likely to influence the spatial distribution of *An. hyrcanus*, two Landsat Enhanced Thematic Mapper (ETM+) images from dry (July 21, 2001) and wet (October 25, 2001) seasons were used to identify and map the landscape units within the study area (Figure 1). The characteristics of this sensor allow the whole study area (70 × 40 km) to be covered in one scene with a spatial resolution of 30 meters. A supervised object-oriented classification was carried out to produce the land-cover map. July's image was first segmented into homogeneous polygons (objects) (Definiens-imaging eCognition™ software). A training dataset of sites of known land cover (sites identified in the field in 2004 and from existing vegetation maps, 2001 [43,44]) was then used to classify each object. The image from the autumn period was processed to map water bodies in order to distinguish the areas flooded by end summer and autumn rainfalls from the ones never flooded. The whole image processing method has been described in [45].

We did not use the reflectance values measured by remote sensing (examples of such an approach for malaria studies in [12]) in order to be able to interpret the results from an ecological point of view. Moreover, the use of land-cover classes instead of reflectance values made it possible to simulate long term land-cover changes.

This map (Figure 2) includes the main biotopes where *An. hyrcanus *larvae and adults were collected: rice fields, reed beds, marshes with *Scirpus*, rush wetland temporarily flooded and clear water.

The presence of *An. hyrcanus *was assumed to be strongly dependent on rice paddies [38,39], and thus the distance to the nearest rice field was computed for each pixel using Geographic Information System (GIS) functionalities (GIS software: ESRI ArcGIS™, Spatial Analyst) (Figure 3).

Finally, the areas where mosquito control measures were applied (EID, unpublished data) were mapped to distinguish rice fields which were not treated with larvicide from those which were. In the "Carbonnière" area, one rice paddy was concerned. No treatments have been performed in the "Marais du Vigueirat", which is a protected natural area.

### Larval population mapping

We used the entomological and environmental data to test for associations between the presence of larvae in breeding sites and land cover variables (biotope and distance to rice field). The presence (at least once over the year) or absence of *An. hyrcanus *larvae in a potential breeding site was the dependant (explained) variable.

We built three logistic regression models using as explanatory variables: i) the biotope, ii) the distance to the nearest rice field, and iii) the simple interaction between i) and ii), as explicative variables. Logistic regression is commonly used to study the relationships between a variable in two modalities (presence/absence) and risk factors which may be qualitative or quantitative variables [46].

The sample size being small (n = 80), a multi-cross-validation procedure was performed in order to assess the stability and accuracy of the models. In the multi-cross-validation procedure, the original sample (here n = 80) is randomly divided into two subsets (n_1 _= 60; n_2 _= 20); models (here logistic regression models) are built from the first sample and the second one is used to generate predicted values (*i.e*., the presence of *An. hyrcanus *larvae, calculated with a given probability threshold) [47]. We used a threshold value of 0.5 [48], which is in correspondence with the optimal cut-off threshold (0.57) estimated by a ROC (Receiver Operating Characteristic) analysis [49]. The accuracy of each model (overall accuracy, sensitivity and specificity [8]) is assessed by comparing the real and predicted values of the second subset. This procedure was repeated 1000 times to test for the stability of the models, to identify the most plausible model and to determine its parameters (R freeware [50]).

Finally, a larval index was computed for each pixel within the image of the study area (ArcGIS Spatial Analyst Tools). The larval index is defined as the probability of observing *An. hyrcanus *larvae in a point at least once during the mosquito season, and is estimated by applying the logistic transformation to each pixel according to its biotope and/or its distance to the nearest rice field.

Two different larval index maps were produced: one with all rice fields (not taking into account mosquito control measures) and one excluding treated rice fields.

### Adult population mapping

Assuming that the abundance of adult mosquitoes depends on the presence of breeding sites in the surroundings [51], we used the larval index map to derive an adult index.

This adult index was defined for each trap location as the mean value of the larval index within a buffer around the trap location. Different buffer sizes (ranging from 100 to 1000 metres) were tested.

The adult index was then compared to the observed abundance of *An. hyrcanus*, to identify the best buffer size. The relationship between the maximum mosquitoes captured and the adult index was established to map the maximum abundance of *An. hyrcanus *adult populations in a given site over the whole study area.

## Results

### Entomological data

*An. hyrcanus *larvae were collected in all the three field study sites ("Carbonnière", "Marais du Vigueirat" and "Méjanes") (Figure 2). Larvae were mostly collected in rice fields (63% of the rice fields were positive sites for *An. hyrcanus*), but were also collected in *Scirpus *marshes (31%) and in a small number of reed beds (1%).

*An. hyrcanus *adult populations were present in two of the field study sites ("Carbonnière" and "Marais du Vigueirat"), with larger densities at "Marais du Vigueirat" than at "Carbonnière" (Figure 3).

*An. hyrcanus *larvae abundance was not spatially auto-correlated among the sites sampled (Moran's I index = 0.06, p = 0.70). Sites were thus considered as spatially independent in the following analysis.

### Larval population mapping

Comparison of the three possible logistic-regression models is shown in Table 1. The model with the best sensitivity/specificity compromise as well as the best overall accuracy explained the presence of larvae as a function of biotope and distance to the nearest rice field (sensitivity = 0.76; specificity = 0.78; overall accuracy = 80%).

**Table 1:** Accuracy assessment of three logistic-regression models for the presence of *Anopheles hyrcanus *larvae, Camargue area, France

Model	% correct	Sensitivity (IC95%)	Specificity (IC95%)
Biotope	77 % (58–89)	0.68 (0.33–1)	**0.85 **(0.71–1)
Distance	69 % (53–.89)	0.74 (0.38–1)	0.71 (0.44–0.92)
Biotope + distance	**80 **% (63–95)	**0.76 **(0.33–1)	0.78 (0.61–0.93)

Distance: Distance to the nearest rice field

The parameters of this model are given in Table 2. The regression coefficients associated with reed beds, rice fields and marshes with *Scirpus *biotopes, as well as with the distance to the nearest rice field were significantly different from zero.

**Table 2:** Regression coefficients of the best model for presence of *Anopheles hyrcanus *larvae, Camargue area, France

Model	Regression coefficient	[95% CI]	p
Intercept	-17.15	[-18.94;-14.86]	-
Distance	-3.21	[-6.17;-1.74]	0.021
Biotope			
Clear water	0	-	-
Rush wetland	0.92	[-0.64;3.03]	0.25
Rice field	17.67	[15.38;19.45]	0.005
Reeds	14.48	[-0.12;16.59]	0.084
Marshes with *Scirpus*	17.50	[14.99;19.24]	0.009

CI: confidence interval

Distance: Distance to the nearest rice field

**Figure 4:**
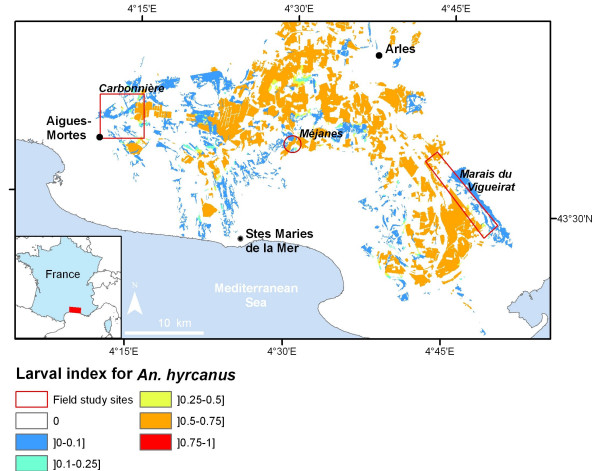
**Anopheles hyrcanus larval index map derived from satellite imagery, Camargue area, France.**

The *An. hyrcanus *larval index map (taking mosquito control into account) is presented in Figure 4. This map highlights the dependence of *An. hyrcanus *on rice paddy areas (Figure 4: areas in orange, probability of presence > 0.5), but also the possibility of eggs being laid in some others biotopes such as marshes with *Scirpus*.

### Adult population mapping

The best correlation between the adult index and the observed maximum number of *An. hyrcanus *captured was observed for a buffer radius of 300 m. These correlations were significant when the mosquito control measures were taken into account in the rice paddies (Pearson r [buffer radius = 300 m] = 0.97, p < 0.05). The optimal size for the buffer around the traps was identified as 300 metres (Table 3).

**Table 3:** Influence of buffer size in the calculation of the *Anopheles hyrcanus *adult index to the correlation coefficient between adult index and real mosquito abundance

**Buffer radius (m)**	**Pearson r**	**p-value**
100	0.63	9.4 10^-3^
200	0.94	6.11 10^-8^
**300**	**0.94**	**3.68 10**^-8^
400	0.83	6.71 10^-5^
500	0.71	2.10 10^-3^
600	0.64	8.15 10^-3^
700	0.59	1.53 10^-2^
800	0.56	2.30 10^-2^
900	0.59	1.71 10^-2^
1000	0.57	2.03 10^-2^

**Figure 5:**
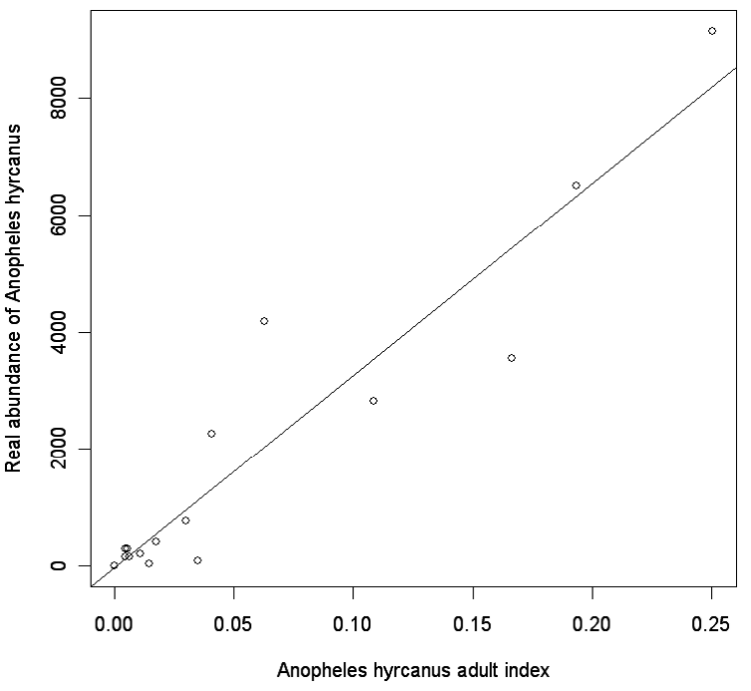
**Bidimensional representation of the real abundanceof *Anopheles hyrcanus *populations in 2005, Camargue area, France versus the adult index derived from satellite imagery**. The circles show the real abundance of *Anopheles hyrcanus *mosquitoes (maximum number of adults trapped in CDC-light traps from March to October) and the solid line is the regression line.

The final relation between the maximum number of *An. hyrcanus *mosquitoes likely to be observed in a given point and the adult index was determined as (Figure 5):

maximum *An. hyrcanus *= 38 918 * adult index [buffer radius = 300 m]

**Figure 6:**
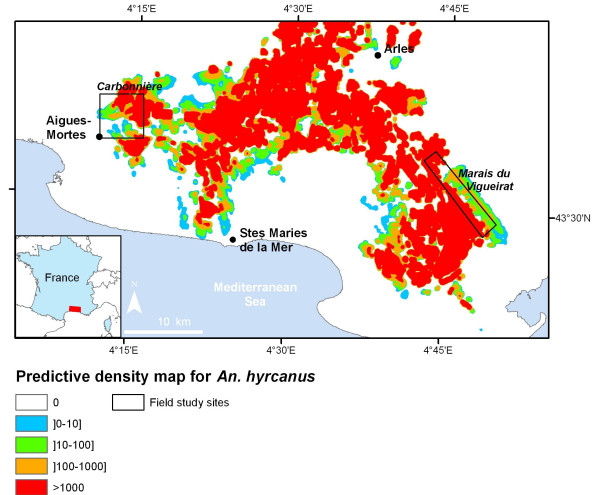
*Anopheles hyrcanus *density map derived from satellite imagery, Camargue area, France.

The final *An. hyrcanus *adult population predictive map is presented in Figure 6. The distribution area of the species includes the breeding sites and their surroundings.

## Discussion

In order to assess the risk of re-emergence of malaria in Europe, a preliminary step is to understand the spatial distribution of potential vector breeding habitats and adult populations. However, carrying out entomological surveys on broad areas remains a heavy task. In this study, remotely sensed data were successfully used to produce predictive maps of larval and adult populations of the main potential malaria vector over a region of southern France, at a scale that provides a locally precise-enough description of the spatial distribution of the vector's abundance, which is an essential component of the risk of emergence of the disease.

Our results highlight a strong link between land cover variables and the presence or absence of *An. hyrcanus *larvae. In particular, the importance of irrigated croplands such as rice fields, which has been described in other contexts [38,39], is confirmed for the Camargue area. The potential of other biotopes such as *Scirpus *marshes to constitute favourable breeding sites for *An. hyrcanus *was also confirmed. This result is of great importance in explaining the presence of this species in the area even when rice paddies are dry (end of summer and autumn). Thus, the final model describing the distribution of *An. hyrcanus *larvae includes both biotope and distance to the rice fields as risk factors. From a biological point of view, this suggests that *An. hyrcanus *females preferentially lay their eggs in rice fields, but also in other biotopes, provided that the latter are close to the main breeding sites (*i.e*., the rice fields). Although these results were already known, this analysis quantifies this relationship, leading to a predictive map of the potential breeding sites for *An. hyrcanus *(Figure 4). An external accuracy assessment of the model revealed a good agreement between observed and predicted values for the larval presence, with both having correct sensitivity and specificity values (Se = 76%; Sp = 78%).

The accuracy of the best logistic regression model is also confirmed by the very strong correlation between the *An. hyrcanus *adult population index, directly derived from the larval index, and field entomological data on adult population abundance (Pearson r = 0.97; p < 0.05). Indirectly, this result can be considered as a second external validation for our larval index map, strengthening the fact that our method is relevant and would also be useful for mapping other mosquito species at high spatial resolutions.

Another result of interest in our study is the determination of the optimal buffer size to compute the adult index (300 metres). This size can be interpreted as the active distance flight of *An. hyrcanus *mosquitoes around the breeding site from which they emerge. A 300 m distance is consistent with ranges usually reported in the literature. Indeed, the active dispersal as appetential flight for mosquitoes usually cover short distances, such as a few hundred meters [52].

This study also stresses the importance of mosquito control measures. When such measures were taken into account, the correlation between the adult index and observed data strongly varied in the "Carbonnière" area but remained unchanged in the natural "Marais du Vigueirat" area, as this area is not treated. Although mosquito control measures in the study area do not target *An. hyrcanus *populations, it seems that the latter are affected.

It was finally possible to map *An. hyrcanus's *adult population due to the linear relationship found between the adult index for a 300 m buffer radius and the real abundance of the adult population (Figures 5 and 6). As the adult index gives an approximation of the number of adult mosquitoes in each pixel, the final map corresponds to a density map for *An. hyrcanus*.

Some limitations of our method must be pointed out. A first weakness of our analysis concerns the time lag between the acquisition of the satellite imagery that was used to map the land cover (2001) and entomological surveys (2005–2006). We assumed that in the study area, land use does not change so much within a 4–5 year duration. Indeed, stakeholders interviewed in October 2006 argued that the land cover tended to stabilize these last years [53]. Between 1991 and 2001, only 3% of the area of the natural regional park of Camargue changed their main class of land cover [43]. In other areas, where important landscape changes take place (deforestation, active urbanization, etc.) [18], the acquisition of the satellite scene has to be concomitant with the field surveys.

In this study, the influence of local parameters (such as water temperature, water depth, substrate type, predators, nutrients and physicochemical such as pH) on mosquito larvae presence was not evaluated. As the main objective was to map *An. hyrcanus *populations, we focused our analysis on environmental variables which could be mapped, *i.e*., which could be easily estimated in each point of the study area. Further studies are nevertheless required to fine-tune the link between local environmental determinants and indices derived from satellite imagery.

Due to the limited adult *An. hyrcanus *population sampling (only 16 sites), it was not possible to directly validate the final abundance map with independent observations. Further entomological surveys are therefore needed to achieve this. Thus, it is essential to verify the hypothesis that there is a correlation between the probabilities of mosquito larvae presences *versus *mosquito adult abundances, by identifying a series of standardized mosquito samples carried out within the Camargue region.

It should also be noted that the final map of larvae presence (the larvae index) indicating the probability of observing *An. hyrcanus *larvae at least once in the year (*i.e*., expressed as an index ranging from 0 to 1), does not correspond to the larval abundance and does not describe the seasonal variations of larvae presence. In the same way, the adult index giving the maximum number of *An. hyrcanus *observed at one time in a year does not describe the temporal dynamics of the adult population. In Camargue, *An. hyrcanus *adult populations are known to increase in June, reach a peak near the middle of August and decrease drastically in September [36]. Thus, to describe and quantify the spatial and temporal distribution of larvae and adult populations, a model for the population dynamics of *An. hyrcanus *has to be developed. This model could integrate the larval index map and meteorological data which drive mosquitos' population dynamics (such as temperature and rainfall) as input.

This study could therefore be considered as the first step in modeling *An. hyrcanus *spatio-temporal dynamics. For instance, the larval index map could be used to derive monthly maps of the potential breeding sites available for *An. hyrcanus *by combining the larval index with data on periods during which sites are flooded (breeding sites are available for *An. hyrcanus *only when they are in water). Then, the dynamics of mosquitoes could be described by a simulation model (agent-based model or a spatial diffusion model [54]).

Another application of this work consists of combining the vector distribution maps with entomological characteristics (life span, trophic preferences, extrinsic incubation period) and host characteristics (human spatial distribution, contact rates) to map *An. hyrcanus *vectorial capacity [55] and the malaria basic reproductive rate (R0) [56]. These indices would help to assess the risk of the emergence of malaria in southern France under different land use changes scenarios, bearing in mind that this risk is considered minimal under the current combination of environmental and socio-economics conditions. Changes in land-cover (increasing rice-field surfaces, urbanization growth, etc) could be easily simulated using GIS, and their impact on the malaria vector's distribution could be calculated using our method.

Moreover, our approach could be combined with other studies mapping *Anopheles *species distributions at a continental scale [41] to derive a multi-scale complementary tool adapted to map the distributions of potential vector species i) at an European scale and ii) more precisely in each risk area (as the Camargue area).

From a broader perspective, it would be pertinent to apply our method to other vector-borne diseases for which the vectors are present in the Camargue, and which have recently re-emerged in Europe, such as the West Nile Virus [45].

## Conclusion

The analysis of the relationships between *An. hyrcanus *larvae presence and environmental indices derived from Landsat imagery allowed us to map the potential breeding sites of this species in the Camargue, a former malaria endemic area in southern France. Based on this larval index, an adult index was calculated, which was strongly correlated with the observed abundance of adult *An. hyrcanus*. This work is a first essential step in assessing the risk of the re-emergence of malaria in this area.

## Authors' contributions

AT carried out the image processing, data analysis and drafted the manuscript. NP contributed to the conception of the study, participated to the entomological field work and participated to results interpretation. CT carried out the entomological field work and identification of mosquitos' species. CL participated in the conception of the study and manuscript revision. HG and DLS contributed in the data analysis and results interpretation. JBF contributed in the entomological field work. FR and SDLR contributed in the conception of the study. DF and TB contributed in the conception of the study, results interpretation and manuscript revision.
